# What Is the Evidence for Cognitive Behavioural Therapy for Insomnia (CBTI) in Improving Sleep in People With Mild Cognitive Impairment or Dementia?

**DOI:** 10.1192/bjo.2024.143

**Published:** 2024-08-01

**Authors:** Charlotte Forbes, Jo Butterworth, Chris Fox, Louise Allan

**Affiliations:** University of Exeter, Exeter, United Kingdom

## Abstract

**Aims:**

There is a well-established association between sleep disturbance and cognitive decline. Poor sleep can have a significant effect on patient and carer wellbeing and is a potentially modifiable risk factor for dementia. Sleep medications are problematic in cognitive impairment due to the increased risk of adverse events such as falls and confusion. There is good evidence for Cognitive Behavioural Therapy for Insomnia (CBTI) in older adults but its effectiveness in cognitive impairment is unclear. In 2021, only one RCT on CBTI in cognitive impairment was identified (Cassidy-Eagle et al. 2018). This review seeks to establish if there is any new evidence.

**Methods:**

Ovid Medline (1946 to present) and clinicaltrials.gov were searched for all interventional trials testing CBTI including RCTs, single-arm studies and protocols, written in English. Inclusion criteria:

1. Adults with a diagnosis of MCI or Alzheimer's dementia;
2. Sleep as a primary outcome, using a validated outcome measure.

Systematic reviews were tracked for references.

**Results:**

172 citations were screened by the first author and 26 underwent full text review. Eight papers were eligible for inclusion. Four of these studied MCI, three looked at people living with dementia (PLWD) and caregivers as a dyad and one combined MCI and Alzheimer's (protocol only).

The search found two pilot RCTs and two protocols for MCI. Cassidy-Eagle et al. (2018) found a highly significant positive effect on four of five sleep outcome measures with large effect sizes. The Insomnia Severity Index (ISI) decreased from 15.29 to 3.25 (p < 0.001; Cohen's d −4.22). Mattos et al. (2021) also found significant improvements on all sleep outcome measures; ISI decreased from 13.5 to 8.3 (p < 0.01).

Three papers study joint CBTI for PLWD and their care partners (one pilot RCT and two protocols). Song et al. (2024) reported improvements in sleep parameters for both participants in the dyad but were not statistically significant. They are recruiting for a larger trial.

**Conclusion:**

This review identified 7 new RCTs in progress. In MCI, new data continue to show a significant association between CBTI and improved sleep. Published data for people with dementia have not found a significant relationship, although the data set remains very limited. It is not yet possible to synthesise the results and future systematic reviews are needed. If effective, CBTI could offer a lower risk alternative to medications in managing sleep disturbance in people with cognitive impairment.

